# Anaphylaxis Management in Acute Care Settings: A Review Based on UK Guidelines

**DOI:** 10.7759/cureus.106669

**Published:** 2026-04-08

**Authors:** SayedaSaaniya SY Mehdi, Munira Khanum

**Affiliations:** 1 Obstetrics and Gynaecology, Conquest Hospital, Hastings, GBR; 2 Gastroenterology, Conquest Hospital, Hastings, GBR

**Keywords:** adrenaline autoinjector, allergy and anaphylaxis, allergy immunology, emergency and acute care, life threatening anaphylaxis, resuscitation council uk

## Abstract

Anaphylaxis, a severe and potentially life-threatening allergic reaction, requires immediate recognition and management within acute care settings. This article reviews current guidelines and practices for anaphylaxis management in the UK, synthesizing evidence from various sources, including the UK Resuscitation Council (2020), National Institute for Health and Care Excellence (NICE) Guidelines (2021), and British Society for Allergy and Clinical Immunology (BSACI) Guidelines (2016), to highlight key recommendations and areas for improvement. The review begins with an overview of the pathophysiology of anaphylaxis, emphasizing the importance of early identification and the initiation of treatment. Current UK guidelines advocate for a systematic approach that includes the immediate use of intramuscular adrenaline as first-line therapy, alongside adjunctive measures such as positioning and airway management. The role of secondary interventions, such as antihistamines and corticosteroids, is also discussed concerning their efficacy and timing. Furthermore, the article addresses the importance of post-anaphylaxis care, including monitoring, patient education, and the provision of adrenaline auto-injectors. Barriers to effective management, such as gaps in clinician training and public awareness, are examined, providing suggestions for enhancing education and readiness. This review underscores the need for a comprehensive and unified approach to anaphylaxis management in acute care, aligning clinical practices with established guidelines to improve patient outcomes. Enhanced awareness and training for healthcare professionals, alongside community engagement, are crucial to minimizing morbidity and mortality associated with anaphylaxis in the UK.

## Introduction and background

Anaphylaxis is an acute, life-threatening systemic allergic reaction that develops rapidly and requires immediate recognition and management. It results from an exaggerated immune response, often immunoglobulin E (IgE)-mediated, triggered by allergens such as foods, medications, insect venom, or latex. Mast cells and basophils release inflammatory mediators, including histamine and leukotrienes, leading to vasodilation, increased vascular permeability, bronchoconstriction, and tissue oedema, potentially progressing to cardiovascular collapse and respiratory compromise if untreated [1-3].

The global incidence of anaphylaxis has increased over recent decades, with population-based estimates ranging from 50 to 112 episodes per 100,000 person-years and a lifetime prevalence of 0.3-5.1% [1]. Despite low case fatality rates (<0.5%), fatalities occur, underscoring the importance of rapid intervention [1,4]. These data highlight anaphylaxis as a common and potentially fatal emergency, with significant implications for clinical practice and healthcare systems.

Clinically, anaphylaxis presents heterogeneously, affecting multiple organ systems. Cutaneous manifestations, such as urticaria and angioedema, are the most frequent, occurring in approximately 80-90% of cases, while respiratory symptoms (~70%), gastrointestinal symptoms (~30-45%), and cardiovascular involvement (~10-45%) are also commonly observed [3]. Diagnosis remains primarily clinical, based on rapid symptom onset following allergen exposure, as no single laboratory test is definitive [1,4].

First-line management universally recommends prompt intramuscular adrenaline, supported by airway management, oxygen, and intravenous fluids as indicated. International guidelines from the European Academy of Allergy and Clinical Immunology (EAACI) and the World Allergy Organization (WAO), alongside UK guidance from the Resuscitation Council UK and the National Institute for Health and Care Excellence (NICE), and the British Society for Allergy and Clinical Immunology (BSACI), emphasise immediate recognition, adrenaline administration, and patient/caregiver education [2,3]. Nevertheless, underuse of adrenaline and delays in administration remain prevalent challenges in both community and hospital settings, contributing to preventable morbidity and mortality [2-4].

Addressing these gaps through clinician education, guideline adherence, and patient awareness is essential in improving outcomes. A thorough understanding of anaphylaxis pathophysiology, clinical presentation, and management strategies is critical to reduce preventable adverse outcomes and optimise patient safety [4,5].

## Review

Methodology

This study employed a narrative literature review to summarize current recommendations for the management of anaphylaxis in acute care settings based on United Kingdom clinical guidelines. Relevant literature published within the past 10-15 years was identified through comprehensive electronic database searches, including PubMed and Google Scholar, as well as established guideline repositories. A systematic approach was used to ensure inclusion of high-quality and clinically relevant sources. Emphasis was placed on guidance from established UK healthcare organizations, including the Resuscitation Council UK, the National Institute for Health and Care Excellence, and the British Society for Allergy and Clinical Immunology, given their authoritative role in shaping evidence-based clinical practice within the UK.

Additional information was obtained from clinical resources provided by the National Health Service, the British National Formulary, and educational materials from Anaphylaxis UK to ensure a well-rounded and practical perspective. Peer-reviewed articles addressing the epidemiology, pathophysiology, clinical presentation, and management of anaphylaxis were also included to enhance the breadth and depth of the review. Where appropriate, recent systematic reviews and consensus statements were prioritised to reflect current best practices. The selected literature was critically appraised and synthesized to provide a clear, structured, and comprehensive overview of evidence-based practices for the recognition and emergency management of anaphylaxis in acute healthcare settings, with particular relevance to frontline clinical care.

Pathophysiology and clinical features of anaphylaxis

Anaphylaxis is a severe, rapidly evolving systemic hypersensitivity reaction that can result in life-threatening airway, respiratory, or circulatory compromise. It most commonly occurs through an immunoglobulin E (IgE)-mediated mechanism in which exposure to an allergen triggers cross-linking of IgE antibodies bound to high-affinity receptors on mast cells and basophils, leading to rapid cellular activation and degranulation [5,6]. This immunological response represents a type I hypersensitivity reaction and typically occurs within minutes of allergen exposure, although delayed presentations have also been reported.

Clinically, the diagnosis of anaphylaxis is based on recognised criteria rather than isolated symptoms, as outlined in guidelines from the Resuscitation Council UK, National Institute for Health and Care Excellence, and British Society for Allergy and Clinical Immunology. These include the acute onset of illness with involvement of the skin and/or mucosal tissue along with respiratory compromise or reduced blood pressure, or the presence of two or more systems involved (e.g., skin, respiratory, cardiovascular, gastrointestinal) following exposure to a likely allergen, or isolated hypotension after exposure to a known allergen [5,6].

This process results in the release of a wide range of inflammatory mediators, including histamine, tryptase, leukotrienes, prostaglandins, and cytokines [7,8]. Histamine contributes to vasodilation and increased vascular permeability, while leukotrienes and prostaglandins are involved in bronchoconstriction and ongoing inflammation. Serum tryptase, a marker of mast cell activation, may be elevated during anaphylaxis and can support the diagnosis; however, it has limited sensitivity and is not required for diagnosis, which remains primarily clinical [7,9]. Tryptase measurement is most useful when obtained within a few hours of symptom onset and may aid in retrospective confirmation in uncertain cases rather than guiding acute management.

These pathophysiological changes produce the characteristic clinical manifestations of anaphylaxis, which commonly involve the skin, respiratory, and cardiovascular systems. Cutaneous features include urticaria, pruritus, flushing, and angioedema, which are often early indicators of the reaction [5,7]. Respiratory symptoms may range from nasal congestion and throat tightness to bronchospasm, wheezing, dyspnoea, and potentially life-threatening upper airway oedema [7,10]. Cardiovascular involvement can lead to hypotension, tachycardia, dizziness, syncope, and progression to shock due to widespread vasodilation and fluid extravasation [5,10].

Gastrointestinal symptoms such as abdominal pain, nausea, vomiting, and diarrhoea may also occur, particularly in food-induced anaphylaxis [10]. In severe cases, patients may experience neurological symptoms such as confusion or a sense of impending doom [7]. The severity and combination of symptoms can vary, and not all clinical features may be present in every case.

In some instances, anaphylaxis may occur through non-IgE-mediated mechanisms, including complement activation or direct mast cell activation by certain medications and contrast agents, such as radiocontrast media, opioids (e.g., morphine), neuromuscular blocking agents (e.g., atracurium), and other drugs, including vancomycin and fluoroquinolones [8-10]. Regardless of the underlying pathway, the final outcome involves widespread mediator release leading to systemic inflammatory responses, with potential for rapid cardiovascular collapse if not treated promptly [6,7].

Epidemiology and prevalence

Anaphylaxis is increasingly recognized as a significant global health concern, with rising incidence reported in many developed countries. Population-based studies estimate the lifetime prevalence of anaphylaxis to range between 0.05% and 2% of the general population [11,12]. In Europe, the estimated lifetime prevalence is approximately 0.3%, with incidence rates ranging from 1.5 to 7.9 cases per 100,000 people every year [11,13]. Although these figures may vary depending on study design and diagnostic criteria, they highlight the growing burden of anaphylaxis in both community and hospital settings.

The most common triggers vary by age group; food allergens are the predominant cause in children, whereas medications, including antibiotics (particularly beta-lactams and penicillins), nonsteroidal anti-inflammatory drugs such as aspirin, anaesthetic agents, and radiocontrast media, are more frequently implicated in adults, along with insect venom [5,14]. Common food triggers include peanuts, tree nuts, milk, and shellfish. Drug-induced reactions are often associated with antibiotics, particularly penicillins, and nonsteroidal anti-inflammatory drugs. Geographic variation also exists, with food-related anaphylaxis being more common in Western countries, whereas insect venom and drug-related anaphylaxis are more frequently reported in parts of Europe and Asia, influenced by environmental exposure, dietary patterns, and healthcare practices.

Hospital admissions for anaphylaxis have increased over recent decades, possibly reflecting improved recognition, reporting, and changes in environmental or dietary exposures [13,15]. Increased awareness among healthcare professionals and the public, as well as better access to emergency services, may also contribute to this observed rise. Despite this increasing incidence, mortality from anaphylaxis remains relatively low and is estimated at fewer than one death per million individuals annually [14,15]. Nevertheless, fatalities can occur rapidly in severe anaphylaxis, particularly in the context of respiratory compromise, underscoring the importance of early recognition and prompt management.

Current UK guideline recommendations for acute in-hospital management of anaphylaxis

In the United Kingdom, acute management of anaphylaxis is guided by recommendations from the Resuscitation Council UK, National Institute for Health and Care Excellence, and British Society for Allergy and Clinical Immunology. While these guidelines share core principles, including early recognition and prompt administration of intramuscular adrenaline, there are minor differences in diagnostic emphasis, dosing, and follow-up recommendations, which are important for clinical practice.

Anaphylaxis is primarily a clinical diagnosis, based on the acute onset of symptoms involving airway, breathing, or circulation (ABC), often with associated skin or mucosal changes. Diagnostic criteria across guidelines are broadly aligned, requiring multisystem involvement or isolated life-threatening airway, respiratory, or circulatory compromise following allergen exposure [1,3,5]. Severity may be graded from mild to severe, with severe anaphylaxis characterised by airway obstruction, bronchospasm, or hypotension leading to shock [2,4]. 

Initial management follows the Airway, Breathing, Circulation, Disability, Exposure (ABCDE) approach, enabling rapid identification and treatment of life-threatening features [7]. Intramuscular adrenaline administered into the anterolateral thigh is the first-line treatment in all guidelines, due to its rapid absorption and effectiveness in reversing vasodilation, bronchospasm, and mucosal oedema [16,17]. However, dosing recommendations vary slightly: most UK guidance, including Resuscitation Council UK, recommends 0.5 mg IM in adults, repeated every five minutes if needed, whereas some international recommendations and auto-injector dosing strategies use 0.3 mg, particularly in community or pre-hospital settings [2-5]. Despite these differences, early administration is prioritised over exact dosing, and delays are consistently associated with worse outcomes [7,16].

Supportive measures, including high-flow oxygen, intravenous fluids, antihistamines, and corticosteroids, may be used as adjuncts but must not delay adrenaline administration [16]. Corticosteroids are typically given as a single acute dose (e.g., hydrocortisone 200 mg IV in adults or weight-based dosing in children), with no routine continuation, and their role remains controversial due to limited evidence in preventing biphasic reactions [4,6]. Patients require close monitoring, involving repeated assessment of vital signs, including blood pressure, heart rate, respiratory rate, oxygen saturation, and level of consciousness, typically every 5-15 minutes initially until stabilisation [16]. Patients with severe or unstable anaphylaxis, or those requiring repeated adrenaline doses, should be managed in a high-dependency or resuscitation setting, whereas stable patients may be monitored on a regular ward with appropriate escalation if deterioration occurs. Observation for biphasic reactions is recommended for four to six hours in mild cases and up to 12-24 hours in severe cases [4,16].

While NICE guidance focuses primarily on post-acute care, including referral and follow-up, the Resuscitation Council UK provides detailed acute management algorithms, making it the most practical framework for frontline emergency care. Overall, clinicians should adopt a structured ABCDE-based approach with early intramuscular adrenaline as the cornerstone of treatment, while being aware of minor variations across guidelines.

Barriers to effective management

Despite clear clinical recommendations, several barriers continue to limit effective management of anaphylaxis. Delayed recognition of symptoms is common, particularly when early manifestations are mild or confined to cutaneous features such as urticaria or angioedema. In isolation, these features do not constitute anaphylaxis; however, they may precede progression to multisystem involvement, where prompt administration of adrenaline is critical [17-19]. Misinterpretation of early signs and diagnostic uncertainty may therefore contribute to delayed treatment and worse outcomes.

Healthcare professionals may also underestimate the severity of reactions, leading to delayed or inappropriate administration of adrenaline. This may include failure to administer adrenaline when indicated, delayed administration, or use of incorrect dosing or route [18]. Limited availability of adrenaline auto-injectors and insufficient patient education further contribute to suboptimal treatment [14,19]. In some cases, patients may not have immediate access to prescribed medication or may lack confidence in recognising symptoms that warrant its use.

In addition, inconsistent adherence to guidelines and variability in clinical practice across healthcare settings may hinder standardised care [13,18]. Differences in training, resource availability, and clinical experience contribute to this variability. Addressing these barriers requires improved awareness, structured training programs, adherence to guideline-based criteria for diagnosis and treatment, and improved access to emergency medications to ensure timely and effective management [16,19].

Several studies have highlighted persistent gaps in the timely and appropriate administration of adrenaline in anaphylaxis. Reported rates of adrenaline administration remain suboptimal, with usage observed in approximately 40-50% of cases in some clinical settings. Delays in administration are also frequently reported and are associated with worse clinical outcomes. In addition, medication errors, including incorrect dosing, inappropriate route of administration, or hesitation in use, have been documented in a proportion of cases, reflecting limitations in training, awareness, and clinical confidence [2,3,5,7]. These findings reinforce the need for improved education, simulation-based training, and adherence to guideline-based management.

Proper use of adrenaline auto-injectors in pre-hospital settings

Adrenaline auto-injectors are essential for the early management of anaphylaxis outside hospital settings. These devices provide rapid intramuscular delivery of adrenaline, which reverses key pathophysiological features such as hypotension and bronchospasm [6,16]. Prompt administration is critical, as delays are associated with poorer outcomes.

Patients are typically prescribed adrenaline auto-injectors in accordance with guidance from organisations such as the Resuscitation Council UK, National Institute for Health and Care Excellence, and British Society for Allergy and Clinical Immunology. Indications include a previous episode of anaphylaxis, particularly where ongoing risk of re-exposure exists, or when risk assessment suggests a high likelihood of future severe reactions (e.g., food allergy, venom allergy, or idiopathic anaphylaxis). In contrast, isolated non-IgE-mediated reactions or mild drug allergies (e.g., uncomplicated diclofenac hypersensitivity without systemic features) do not typically warrant auto-injector prescription unless the risk of progression is considered significant.

Patients with a history of severe allergic reactions are advised to carry auto-injectors at all times. However, studies show that many patients and caregivers have inadequate knowledge of when to administer the device, correct injection technique, and the need for immediate use at the onset of symptoms, leading to delays in treatment [14,20]. This highlights a gap between prescription and effective use in real-life situations. Regular training, repeated demonstrations, and patient education are therefore critical to ensure timely administration during allergic emergencies. Education should emphasise early recognition of anaphylaxis, confidence in device use, and the importance of immediate administration without hesitation [16,20].

Adrenaline auto-injectors should be administered immediately at the first signs of anaphylaxis involving airway compromise, breathing difficulty, or circulatory symptoms, rather than waiting for complete symptom progression, and patients should be advised to seek emergency medical attention after use.

Post-anaphylaxis care and follow-up

Appropriate follow-up care after an anaphylactic episode is essential for preventing recurrence and improving long-term outcomes. Patients should be observed for four to six hours following symptom resolution for mild reactions and up to 12-24 hours for severe reactions or those requiring repeated doses of adrenaline, in accordance with UK and international guidelines, to monitor for biphasic reactions [7,16]. Referral to an allergy specialist is recommended to identify triggers, confirm the diagnosis, and develop a personalized management plan [19]. Follow-up care should also include education on allergen avoidance, emergency action plans, and correct use of adrenaline auto-injectors [14,16]. These measures help reduce recurrence risk and improve patient preparedness.

Psychological impact of anaphylaxis

Anaphylaxis can have substantial psychological effects on patients and their families. Individuals who have experienced severe allergic reactions often report anxiety, fear of recurrence, and reduced quality of life [21]. Children with food allergies and their caregivers may experience significant stress related to accidental exposure in school or social settings [21,22]. These psychological factors may lead to lifestyle restrictions, heightened vigilance, and avoidance behaviours that can impact daily functioning. Integrating psychological support and patient counselling into long-term care may therefore help address these concerns and improve overall wellbeing [21].

Role of training and healthcare education

Training and education of healthcare professionals play a critical role in improving anaphylaxis management. Simulation-based training and structured clinical protocols- defined as standardised, stepwise clinical pathways or guideline-based algorithms that guide assessment and management- have been shown to enhance clinicians’ ability to recognise symptoms and initiate prompt treatment [18]. Continuing medical education programs can also improve adherence to clinical guidelines and increase confidence in the use of adrenaline auto-injectors [17]. Education should extend beyond hospital settings to include primary care providers, paramedics, teachers, and caregivers who may encounter allergic emergencies [22]. Such multidisciplinary training initiatives can contribute to earlier intervention and improved patient outcomes.

Community engagement and public awareness

Public awareness and community engagement are important components of anaphylaxis prevention and management. Educational campaigns can improve recognition of allergic reactions and encourage timely emergency response [22]. Schools and community institutions increasingly implement allergy management policies and maintain accessible epinephrine auto-injectors for individuals at risk [19]. Training teachers, caregivers, and community members in recognizing anaphylaxis and administering adrenaline may significantly reduce delays in treatment during emergencies and improve survival outcomes.

Recent advances in research

Recent advances in allergy research have improved understanding of anaphylaxis mechanisms and management strategies. Molecular allergology techniques now allow more precise identification of allergen components and improved risk stratification [5]. Biomarkers such as serum tryptase, a mast cell-derived protease released during anaphylaxis, are increasingly used as an adjunct to support diagnosis, although they are not required to initiate treatment [9]. Tryptase levels typically peak one to two hours after symptom onset and are best interpreted when measured acutely and compared with a baseline level, with an elevated level supporting mast cell activation; however, a normal result does not exclude anaphylaxis. Therefore, clinical management remains primarily diagnosis-based and should not be delayed for laboratory confirmation, though serial measurements may have a limited role in supporting diagnosis rather than monitoring recovery. In addition, emerging therapies targeting immunologic pathways involved in allergic inflammation are being explored as potential strategies for preventing severe allergic reactions [5,6]. These developments may contribute to more personalised and targeted approaches to anaphylaxis management in the future.

Future directions

Future strategies for improving anaphylaxis care should focus on early recognition, broader availability of adrenaline auto-injectors, and improved adherence to clinical guidelines. Advances in diagnostic technologies may help identify individuals at higher risk of severe reactions [5]. Development of more user-friendly auto-injector devices and expansion of public education initiatives may further reduce delays in treatment and improve outcomes in acute settings [20]. Strengthening epidemiological surveillance systems and promoting multidisciplinary collaboration will also be important for improving prevention and management strategies worldwide.

## Conclusions

Anaphylaxis is a rapidly progressive, life-threatening condition that requires prompt recognition and immediate treatment to prevent serious morbidity and mortality. Despite the availability of well-established national and international guidelines, including those from the Resuscitation Council UK, National Institute for Health and Care Excellence, and British Society for Allergy and Clinical Immunology, which emphasise early recognition, prompt intramuscular adrenaline administration, and structured follow-up care, delays in diagnosis and treatment remain a significant concern in acute care settings. Studies have shown that adrenaline is underused in a substantial proportion of cases, with reported rates approaching 50%, and delayed administration is associated with worse outcomes and preventable fatalities.

Effective post-anaphylaxis care, including specialist referral, patient education, and appropriate follow-up, plays a crucial role in preventing recurrence and improving long-term outcomes. However, barriers such as inadequate training, lack of awareness, inconsistent guideline implementation, and limited access to adrenaline auto-injectors continue to impact optimal care delivery. These gaps likely reflect variability in clinician experience, insufficient dissemination and uptake of guidelines, and challenges in recognising early or atypical presentations.

Addressing these challenges requires targeted interventions, including structured training programmes, improved dissemination and implementation of guidelines, increased public awareness, and better access to emergency medications. Strengthening adherence to evidence-based recommendations and reinforcing early recognition and timely administration of adrenaline are essential. A multidisciplinary and patient-centered approach remains key to improving outcomes in both hospital and community settings.
